# Divergent Cytochrome *c* Maturation System in Kinetoplastid Protists

**DOI:** 10.1128/mBio.00166-21

**Published:** 2021-05-04

**Authors:** Asma Belbelazi, Rachel Neish, Martin Carr, Jeremy C. Mottram, Michael L. Ginger

**Affiliations:** a School of Applied Sciences, University of Huddersfield, Huddersfield, United Kingdom; b York Biomedical Research Institute, University of York, York, United Kingdom; c Department of Biology, University of York, York, United Kingdom; University of Georgia

**Keywords:** cytochrome *c*, *Leishmania*, mitochondrial metabolism, posttranslational modification (PTM), protist, *Trypanosoma brucei*, posttranslational modification, protists

## Abstract

In eukaryotes, heme attachment through two thioether bonds to mitochondrial cytochromes *c* and *c*_1_ is catalyzed by either multisubunit cytochrome *c* maturation system I or holocytochrome *c* synthetase (HCCS). The former was inherited from the alphaproteobacterial progenitor of mitochondria; the latter is a eukaryotic innovation for which prokaryotic ancestry is not evident. HCCS provides one of a few exemplars of *de novo* protein innovation in eukaryotes, but structure-function insight of HCCS is limited. Uniquely, euglenozoan protists, which include medically relevant kinetoplastids *Trypanosoma* and *Leishmania* parasites, attach heme to mitochondrial *c*-type cytochromes by a single thioether linkage. Yet the mechanism is unknown, as genes encoding proteins with detectable similarity to any proteins involved in cytochrome *c* maturation in other taxa are absent. Here, a bioinformatics search for proteins conserved in all hemoprotein-containing kinetoplastids identified kinetoplastid cytochrome *c* synthetase (KCCS), which we reveal as essential and mitochondrial and catalyzes heme attachment to trypanosome cytochrome *c*. KCCS has no sequence identity to other proteins, apart from a slight resemblance within four short motifs suggesting relatedness to HCCS. Thus, KCCS provides a novel resource for studying eukaryotic cytochrome *c* maturation, possibly with wider relevance, since mutations in human HCCS leads to disease. Moreover, many examples of mitochondrial biochemistry are different in euglenozoans compared to many other eukaryotes; identification of KCCS thus provides another exemplar of extreme, unusual mitochondrial biochemistry in an evolutionarily divergent group of protists.

## OBSERVATION

Trypanosomatid parasites of the genera *Trypanosoma* and *Leishmania* are responsible for a variety of serious neglected tropical diseases and belong to the class of flagellate protists called Kinetoplastea. Numerous aspects of kinetoplastid mitochondrial biology, including genome organization (1), RNA editing, protein/tRNA import (2), and cristae formation (3) are highly divergent or unique compared with many eukaryotes.

A fundamental aspect of mitochondrial function is covalent attachment of heme to mitochondrial cytochromes *c* and *c*_1_ within the mitochondrial intermembrane space. For this posttranslational modification, thioether bonds form between heme vinyl groups and cysteine sulfhydryl groups of a CxxCH heme-binding motif within the apocytochrome. The stereochemistry of this heme attachment is conserved across evolution: the 2-vinyl group of heme attaches to the first cysteine, the 4-vinyl group attaches to the second cysteine, and histidine provides an axial ligand to the heme iron. In most eukaryotes, holocytochrome *c* synthetase (HCCS), associated with the outer leaflet of the mitochondrial inner membrane, catalyzes heme attachment to mitochondrial cytochromes *c* (4). In the protomitochondrion, however, the multisubunit, integral membrane cytochrome *c* maturation system I provided an ancestral pathway for *c*-type cytochrome biogenesis. It is retained, partially mitochondrially encoded, in many plants and a few protists. Instances of eukaryotes containing both maturation systems are extremely rare: orphan taxon and predatory flagellate Ancoracysta twista reportedly contains HCCS and system I but is now extinct in the laboratory (5), and a survey of the 1000 plant transcriptome resource (6) suggests that club mosses Phylloglossum drummondii and Huperzia squarrosa possess HCCS plus nuclear gene-encoded system I fragments or a mitochondrial CcmF pseudogene, respectively (A. Belbelazi, M. Carr, and M. L. Ginger, unpublished observations).

Kinetoplastid protists are the only eukaryotes where mitochondrial cytochromes are present but evidence of a cytochrome *c* maturation system is absent (4, 7, 8). Moreover, kinetoplastids and other euglenozoans (e.g., Euglena gracilis) are unique in that heme is bound through only a single thioether linkage in mitochondrial cytochromes *c*. In Euglenozoa, AAQ**C**H and FAP**C**H are the conserved heme-binding motifs in cytochromes *c* and *c*_1_, respectively (the residue at the proximal heme-binding cysteine in normal *c-*type cytochromes is underlined; the heme-binding cysteine conserved in all cytochromes *c* is shown in bold type). Why euglenozoans possess mitochondrial cytochromes *c* with heme bound by a single thioether bond is a mystery of almost 50 years standing. No noticeable difference in the physicochemical properties of euglenozoan cytochromes *c* is known (9). Yet, the activities of *Euglena* cytochrome *c* reductase and oxidase vary, depending on the source of the cytochrome *c* used plus there are fitness costs in trypanosomes engineered to express only CxxCH heme-binding cytochrome *c* (10). This leaves it possible that single cysteine linkage affects electron transport through the mitochondrial respiratory chain (11). The strict conservation of phenylalanine and proline within heme-binding motifs of kinetoplastid and *Euglena* cytochrome *c*_1_ is another puzzle and potentially unique. Perhaps, the proline introduces a local bend in the polypeptide that allows accommodation of the phenylalanine side chain within the protein’s tertiary structure (11).

**FIG 1:**
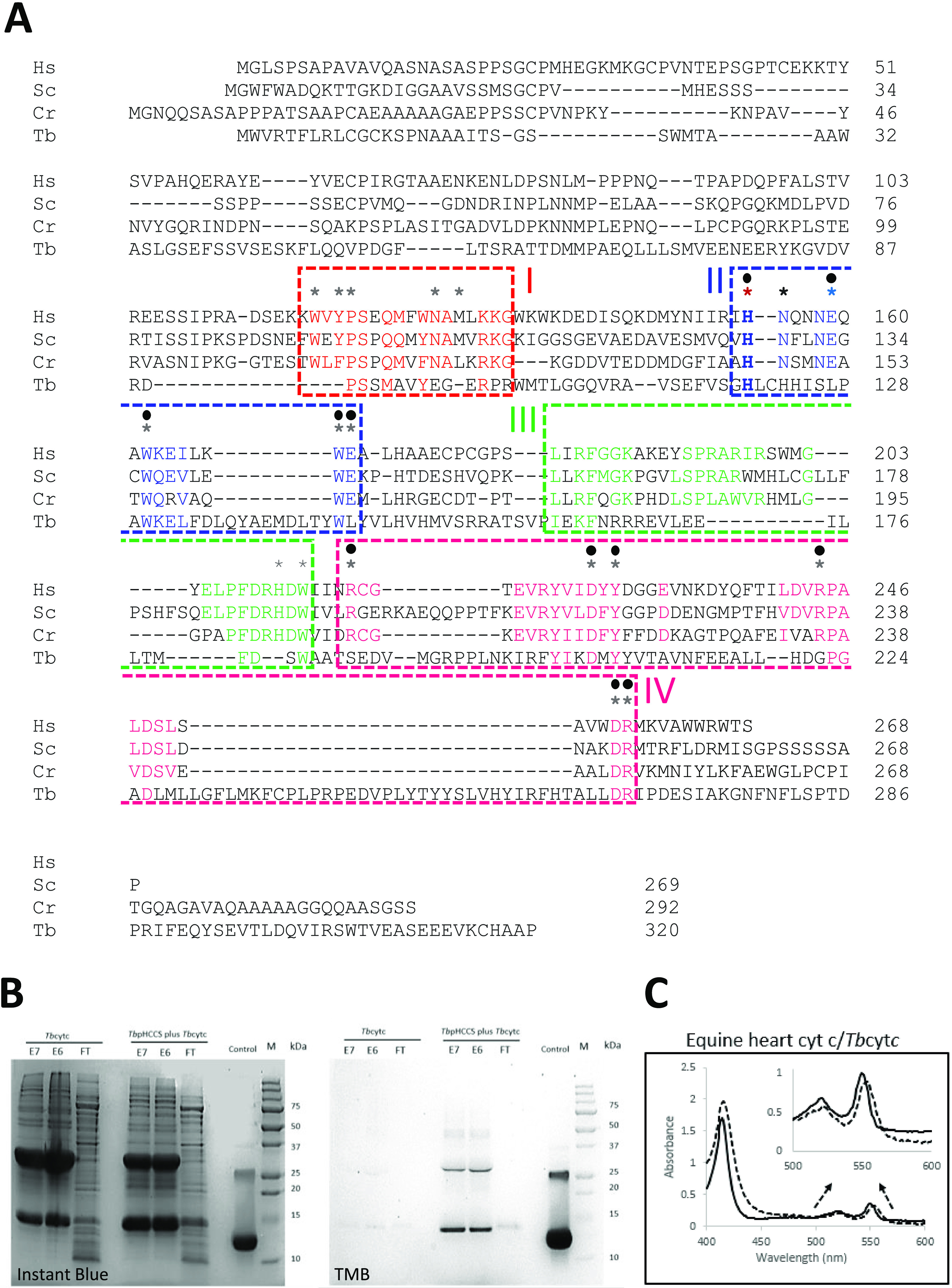
(A) KCCS is a novel protein exhibiting very little sequence similarity to HCCS. MAFFT was used to align T. brucei (Tb) KCCS with HCCS from diverse taxa. HCCS-defining motifs I to IV are boxed. His154 is denoted by a red asterisk. Mutations seen in MLS patients are indicated by blue asterisks. Gray asterisks indicate residues analyzed by site-directed mutagenesis of Homo sapiens (Hs) HCCS (10, 21). Circles denote where site-directed mutation decreased HCCS activity. Cr, Chlamydomonas reinhardtii (XP_001697002.1); Hs, Homo sapiens (NP_001116080.1); Sc, Saccharomyces cerevisiae (NP_009361.1). NCBI reference sequences are provided in parentheses in the preceding sentence. (B and C) *Tb*KCCS-catalyzed maturation of trypanosome cytochrome *c* (*Tb*CYTC). (B) Soluble fractions from E. coli induced for expression (described in Text S1) of either His_6_-tagged *Tb*CYTC or His_6_-tagged *Tb*CYTC plus *Tb*KCCS from pCDFDuet-1 (Novagen) were purified by Ni^2+^ affinity chromatography. Flowthrough (FT) and elution fractions (E6 and E7) from each culture were subjected to acetone precipitation and analyzed by SDS-PAGE under nonreducing conditions. Duplicate 12% gels were stained with either Instant Blue (to confirm protein loading) or 3,3′,5,5′-tetramethylbenzidine (TMB) (to detect covalent attachment of heme to protein). Fifty nanograms of equine holocytochrome *c* was loaded in the control lane of both gels. Dimerization of cytochrome *c*, evident in all lanes is explained by “domain-swapping” of the C-terminal α-helix (27) (Text S1). (C) Pyridine hemochrome spectra for recombinant trypanosome (dashed line) or equine heart cytochrome *c* (solid line) were recorded at 25°C following disodium dithionite addition. The concentration of cytochromes analyzed was 13 μM. The spectra were normalized by Soret band intensity. The inset expands the 500- to 600-nm region of the two spectra, indicating the diagnostic α-band maximum at 553 nm for cytochrome *c* with heme bound by a single thioether bond, red-shifted relative to the 550-nm α-band maximum for cytochrome *c* with a CxxCH heme-binding motif. Instant blue- and TMB-stained gels of the purified cytochrome preparation used for spectroscopy are shown in Fig. S2 in the supplemental material.

To resolve how kinetoplastids mature their unique mitochondrial *c*-type cytochromes, we sorted candidate mitochondrial proteins to identify those conserved in all kinetoplastids, except for plant-pathogenic *Phytomonas*. In *Phytomonas*, adaptation to carbohydrate-rich plant latex correlates with secondary loss of mitochondrial cytochromes and other hemoproteins (12); thus, we reasoned that in *Phytomonas*, a cytochrome *c* maturation system would also be lost. Candidate mitochondrial proteins were then screened for motifs similar to any present in proteins belonging to the four biogenesis systems known to catalyze heme attachment to a cysteine sulfhydryl (see Text S1 in the supplemental material). We identified a single hypothetical protein, highly conserved across the Kinetoplastea (see Fig. S1 in the supplemental material) but absent from *Phytomonas* (encoded by Tb927.3.3890 in Trypanosoma brucei; LmxM.08_29.1300 in Leishmania mexicana) that exhibited colinearity but very limited sequence similarity to four HCCS motifs required for thioether bond formation (Fig. 1A). Similarity between this kinetoplastid protein and human HCCS was too limited to be detected by PSI-BLAST, but the histidine (His154) essential in HCCS for heme attachment to apocytochrome *c* (13) was present at an analogous position in candidate kinetoplastid cytochrome *c* synthetase (KCCS).

10.1128/mBio.00166-21.1 TEXT S1 The complete description of methods covers the following: (i) bioinformatics; (ii) biochemical validation of KCCS candidature (including details of plasmid construction, recombinant protein expression and purification, analysis of cytochrome *c* maturation by SDS-PAGE, 3,3′,5,5′-tetramethylbenzidine-staining, and UV/visible [UV/VIS] spectroscopy); (iii) explanation of the cytochrome *c* dimerization seen in Fig. 1B; (iv) genetic manipulation of Leishmania mexicana; (v) analysis of CRISPR-Cas9 L. mexicana mutants by flow cytometry; and (vi) analysis of KCCS localization by fluorescence microscopy. Download Text S1, DOCX file, 0.04 MB. Copyright © 2021 Belbelazi et al. 2021 Belbelazi et al. https://creativecommons.org/licenses/by/4.0/ This content is distributed under the terms of the Creative Commons Attribution 4.0 International license.

10.1128/mBio.00166-21.2 FIG S1 KCCS is a conserved kinetoplastid protein. Ad, *Angomonas deanei* (EPY32355.1); Bs, *Bodo saltans* (CUF09763.1); Lm, Leishmania mexicana (XP_003872427.1); Pk_sp, *Perkinsela* (KNH04224.1); Tb, Trypanosoma brucei (XP_843981.1); Tbrr, *Trypanoplasma borreli* (retrievable by BLAST analysis from accession GHOB01000228.1). Genbank accession numbers for the sequences used in the alignment are provided in the parentheses. Download FIG S1, PDF file, 0.3 MB. Copyright © 2021 Belbelazi et al. 2021 Belbelazi et al. https://creativecommons.org/licenses/by/4.0/ This content is distributed under the terms of the Creative Commons Attribution 4.0 International license.

10.1128/mBio.00166-21.3 FIG S2 Purity of and covalent heme attachment to *Tb*^His^CYTC purified for UV/VIS spectroscopy. *Tb*^His^CYTC was purified from 8 liters of E. coli induced for recombinant expression of *Tb*^His^CYTC and *Tb*KCCS as described in Text S1 and concentrated to 0.5 ml using a Vivaspin-20 centrifugal concentrator with a molecular weight (m.w.) cutoff of 3 kDa. A portion (1/250th) of the purified protein was taken without acetone precipitation for analysis by SDS-PAGE under nonreducing conditions. Duplicate 12% polyacrylamide gels were stained with either Instant Blue (to confirm purity) or 3,3′,5,5′-tetramethylbenzidine (to detect covalent attachment of heme to protein). Equine holocytochrome *c* was loaded as indicated. Download FIG S2, PDF file, 0.5 MB. Copyright © 2021 Belbelazi et al. 2021 Belbelazi et al. https://creativecommons.org/licenses/by/4.0/ This content is distributed under the terms of the Creative Commons Attribution 4.0 International license.

To assess KCCS candidature, we coexpressed recombinant Tb927.3.3890 and T. brucei cytochrome *c* in Escherichia coli. We reported previously that T. brucei apocytochrome *c* is neither subject to spontaneous maturation in the E. coli cytoplasm nor a substrate for the endogenous periplasmic E. coli cytochrome *c* maturation system (which is expressed minimally under the aerobic conditions we used to cultivate our E. coli cultures) (7). Here, recombinant expression of T. brucei cytochrome *c* bearing an N-terminal hexahistidine tag also resulted in no detectable holocytochrome *c* formation. Coexpression of Tb927.3.3890 and His_6_-tagged T. brucei cytochrome *c*, however, resulted in heme attachment to the latter, as shown by sodium dodecyl sulfate-polyacrylamide gel electrophoresis (SDS-PAGE) of purified protein and staining for covalently bound heme (Fig. 1B). Pyridine hemochrome spectra of purified recombinant trypanosome holocytochrome *c* confirmed heme attachment via a single thioether bond (Fig. 1C): in the spectra shown, the pyridine hemeochrome α-band maximum of the recombinant cytochrome was 553 nm, and clearly red-shifted in comparison with the corresponding 550-nm α-band maximum of cytochromes *c*, which bind heme via two thioether bonds (equine cytochrome *c* in Fig. 1C). Thus, Tb927.3.3890 is appropriately referred to as T. brucei *KCCS* (Tb*KCCS*).

**FIG 2:**
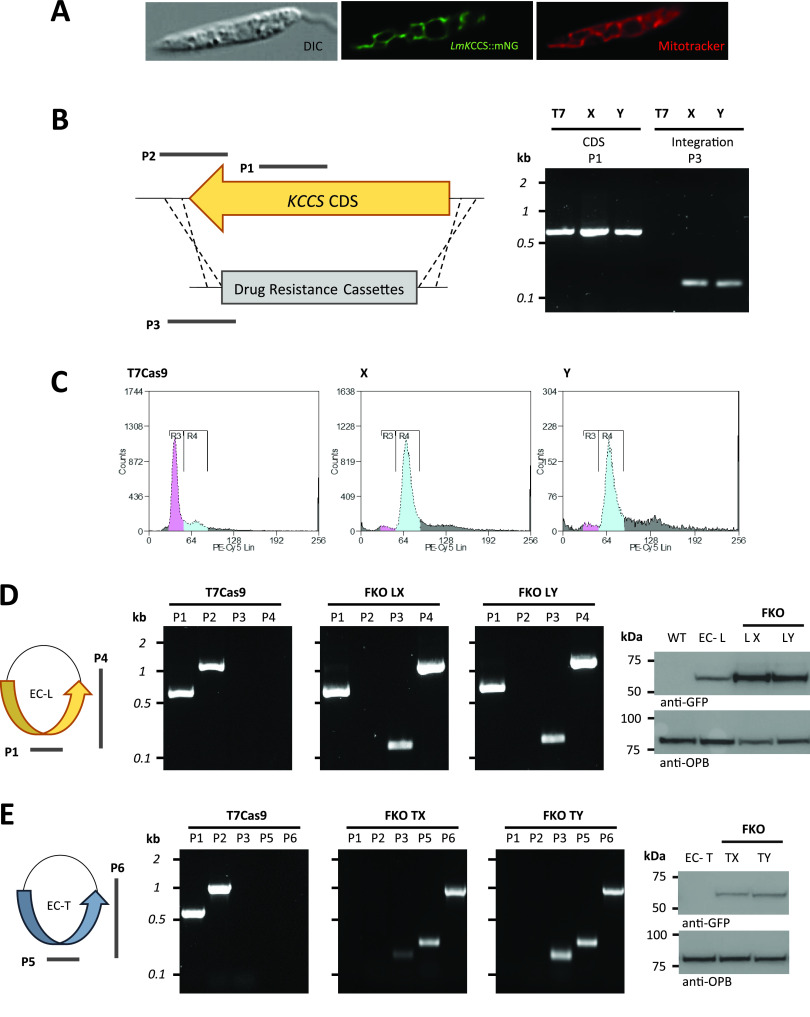
(A) Mitochondrial localization of Lm*KCCS*::mNeonGreen in live, CyGEL-immobilized L. mexicana. DIC, differential interference contrast. (B to E) CRISPR-Cas9 genome editing of L. mexicana reveals that Lm*KCCS* is an essential gene. (B) Homologous recombination of drug resistance cassettes into Lm*KCCS* loci with amplicons from PCR-mapping indicated. P1, amplicon within the Lm*KCCS* coding sequence (CDS); P2, downstream and within Lm*KCCS*; P3, downstream of Lm*KCCS* and within *PUR* or *BSD* resistance cassettes. Diagnostic PCR from genomic DNA (gDNA) templates extracted from *Leishmania* populations (X and Y) after transfection with single guide DNA (sgDNA) and template donor DNA for CRISPR-Cas9 gene editing. (C) Analysis of DNA content in propidium iodide-stained cell populations T7Cas9, X, and Y by flow cytometry. (D and E) Episomal expression of Lm*KCCS*::*GFP* (EC-L) with PCR amplicons for P1 and P4 (D) or episomal expression of Tb*KCCS*::*GFP* (EC-T) and PCR amplicons for P5 and P6 indicated (E). Also shown, PCR mapping of T7Cas9 parental L. mexicana and facilitated knockout (FKO) clones LX and LY (D) or TX and TY (E) together with immunoblot analysis of *Lm*KCCS::GFP or *Tb*KCCS::GFP expression in wild-type L. mexicana, episome-transfected L. mexicana, and FKO clones. For the loading control on the immunoblots, expression of oligopeptidase B (anti-OPB) was detected. WT, wild type.

For molecular genetics analyses of KCCS, we used L. mexicana engineered for tractable CRISPR-Cas9 genome editing (denoted as T7 in Fig. 2B and T7Cas9 in Fig. 2C to E) (14). *Leishmania* promastigotes have no capacity for anaerobic growth; thus, mitochondrial cytochromes are essential (15). Tagged with mNeonGreen and expressed from an endogenous chromosomal locus, L. mexicana *KCCS* (Lm*KCCS*) showed mitochondrial localization (Fig. 2A), consistent with a role in mitochondrial cytochrome *c* maturation. Mitochondrial localization was observed irrespective of whether the mNeonGreen tag was N or C terminal, providing evidence for an internal hydrophilic mitochondrial import signal, as described in yeast HCCS (16). We were unable to generate Lm*KCCS* null mutants and were only able to delete both chromosomal copies of Lm*KCCS* without further genome rearrangements following episomal expression of green fluorescent protein (GFP)-tagged Lm*KCCS* or Tb*KCCS* (Fig. 2B to E). In the absence of pNUS-derived episomes, CRISPR-Cas9-mediated disruption of both Lm*KCCS* alleles in diploid L. mexicana resulted in genome duplication, as well as the site-specific integration of drug resistance cassettes, as revealed by propidium iodide (PI) staining and flow cytometry of methanol-fixed logarithmic phase parasites (Fig. 2C). Thus, CRISPR-Cas9 genome editing indicated that Lm*KCCS* is an essential gene.

Our comparative genomics approach identified KCCS and provides a mechanism by which kinetoplastids mature their mitochondrial cytochromes *c* with heme attached via a single thioether bond. Other than the missing thioether bond, X-ray structures of trypanosomatid and yeast holocytochromes *c* are very similar, although the former (with either AxxCH or an engineered CxxCH heme-binding motif) is a very poor substrate for yeast HCCS (17), presumably because HCCS requires interaction with amino acids upstream of the heme-binding motif (18, 19).

The inability to detect proteins homologous to KCCS outside of the Kinetoplastea suggests that KCCS is more likely to be an extreme or highly divergent HCCS, rather than derived through convergent evolution. Yet, irrespective of its origin, KCCS provides another example of “extreme biology” within a group of protists already well-known for pushing the boundaries (20).

Further characterization should indicate whether the amino acid differences and insertions observed in *Perkinsela* KCCS reflect this taxon’s basal position in kinetoplastid phylogenies or the particular, unusual niche occupied by this obligate symbiont of *Paramoeba* (21). There is also wider significance to our observations: HCCS mutation in humans causes microphthalmia with linear skin lesions (MLS) (22, 23), but insight into HCCS catalysis, and thus the mechanistic consequence(s) of HCCS mutation, are in their infancy (13, 24–26). Our earlier work replacing “AxxCH” cytochrome *c* in T. brucei with a “CxxCH” variant and characterizing the resultant incorrectly matured cytochrome (10) indicates that KCCS is a highly diverged HCCS that cannot act on the first cysteine of a conventional CxxCH heme-binding motif. Indels, deletions, and substitutions within motifs I and II of KCCS, including substitution of the Glu159 MLS mutation (22), may be particularly informative for future study of HCCS catalysis, since these are the motifs believed to mediate heme binding and release (13, 26). Intriguingly, Arg217, the other residue mutated in MLS patients is also not conserved in KCCS.

Detailed methods are available in Text S1 in the supplemental material.
