# Supplementary material for: Divergent Cytochrome c Maturation System in Kinetoplastid Protists
Source: mBio. 2021 May 4;12(3):e00166-21. doi: 10.1128/mBio.00166-21 (PMC8262978; doi:10.1128/mBio.00166-21)
Supplement: TEXT S1 [file mbio.00166-21-s0001.docx]

Extreme divergence of the kinetoplastid cytochrome *c* maturation system

Asma Belbelazi, Rachel Neish, Martin Carr, Jeremy C. Mottram, and Michael L. Ginger

**TEXT S1** Materials and Methods

**Bioinformatics.** KCCS was identified from a bioinformatics sift of proteins present in *T. brucei* mitochondrial proteomes (**1**, **2**). 295 proteins identified as without orthologues in *Phytomonas* were then analysed manually for presence of any possible motifs or similarity to motifs present in any protein previously characterised as involved in any of the four biogenesis systems known to catalyse thioether bond formation between heme and a cysteine sulphydryl (**3**-**6**). Disorder predictions were made using IUPredA and MFDp2 (**7**, **8**). Multiple sequence alignment (MAFFT) was used to align peptide sequences (**9**).

**Biochemical validation of KCCS candidature.** For expression of recombinant cytoplasmic *Tb*HCCS and/or *Tb*CYT*C*, CDSs were sub-cloned into pCDFDuet-1 (Novagen): Tb*CYTC* was sub-cloned into HindIII-EcoRI-digested multiple cloning site 1 (or MCS1), *Tb*HCCS was sub-cloned into XhoI-NdeI-digested multiple cloning site 2 (or MCS2). Forward and reverse primer combinations for PCR amplication of *Tb*HCCS or *Tb*CYT*C*, CDSs, respectively, from genomic DNA templates were tt*gaattc*gcatgccaccaaaggagcgtgc and gc*aagctt*ttagtcctttaatgtctcgagg or cac*catatg*tgggtgaggacattcctgc and aag*ctcgag*tcacggtgccgcatggcattttac. Restriction sites introduced into the primers are italicised. Recombinant protein expression of *Tb*HCCS, His_6_-TbCYT*C*, or *Tb*HCCS and His_6_-*Tb*CYT*C* was induced in *E. coli* Rosetta (Novagen) by addition of 1mM IPTG; induced cultures were allowed to grow for 24 h at 18^o^C at 100 rpm under aerated conditions. *E. coli* cultures were grown in Luria broth (Melford L24400-500.0) without addition of exogenous heme. Following induction of recombinant protein expression, bacterial cells were collected by centrifugation and re-suspended in 20 ml lysis buffer (Tris-HCl (20 mM pH 8.0); NaCl (500 mM); Triton X-100 (0.02% v/v); imidazole (20 mM); glycerol (10% v/v)) per l culture. Protease inhibitor PMSF (100 mM) was immediately added (10 μl per ml of resuspended cells) and the resuspension left shaking (50 rpm, room temperature, 30 min). After this incubation, lysing cells were subject to further disruption by ultra-sonication using a burst frequency of 5 sec on/15 sec off for 10 min at an amplitude of 85%. Following sonication, the suspension was centrifuged at 15 000 x *g* (30 min; 4^o^C) and the supernatant stored at -20^o^C prior to protein purification. Apo- and holocytochromes *c* were purified by Ni^2+^-affinity chromatography using Amintra Ni-NTA resin (1 ml per l culture harvested) under native conditions using wash (Tris-HCl (20 mM pH 8.0); NaCl (300 mM); Triton X-100 (0.02% v/v); imidazole (20 mM); glycerol (10% v/v)) and elution (Tris-HCl (20 mM pH 8.0); NaCl (300 mM); Triton X-100 (0.02% v/v); imidazole (500 mM); glycerol (10% v/v)) buffers. Purified cytochromes were concentrated using a Vivaspin-20 centrifugal concentrator with a m.w. cut-off of 3 kDa Prior to SDS-PAGE proteins were typically subject to acetone precipitation; SDS-PAGE was carried out under non-reducing conditions; and prior to gel-loading samples were heated to 95^o^C for 5 min in loading buffer containing SDS (2%); glycerol (10%); bromophenol blue (0.01%); and Tris-HCl (100 mM pH 6.5). Heme-staining of SDS-PAGE gels using 3,3',5,5'-tetramethylbenzidine (TMB) was carried out as described previously (**10**). Pyridine hemochrome spectra were acquired using a Cary 4000 uv/vis spectrophotometer following the protocol laid out by Barr and Guo (**11**). In our experiments purified recombinant *Tb*CYTC or horse heart cytochrome *c* were soluble in 50 mM Tris-HCl (pH 7.5). To measure oxidized heme spectra, protein solutions were mixed 1:1 with a solution of NaOH (0.2 M), pyridine (40% v/v) and K_3_Fe(CN)_6_ (500 μM) in a final volume of 1 ml; to subsequently measure reduced heme spectra, 10 μl of Na_2_O_4_S_2_ (0.5 M) in 0.5 M NaOH was added to protein/pyridine/ K_3_Fe(CN)_6_ solutions. Difference (reduced minus oxidized) spectra are shown in Fig. 1C.

**Explanation of cytochrome *c* dimers.** From Fig. 1B: oligomerization of mitochondrial cytochrome *c* has been known for almost 60 years (**12**) and is evident in cytochrome *c* preparations from the 1940s (**13**). Dimerization results from displacement of the C-terminal α-helix from monomeric cytochrome *c* and replaced by the corresponding (also displaced) helix from another cytochrome *c* molecule (**14**). This readily explains the dimerization evident in all lanes where cytochrome *c* is present in the Instant Blue-stained gel from Fig. 1B albeit that there is a large proportion of dimer relative to monomer purified from *E. coli* expressing either *Tb*cytc or *Tb*cytc plus *Tb*KCCS. Where *Tb*KCCS is expressed simultaneously with its apo-cytochrome substrate, comparison of TMB- and Instant Blue-stained gels indicates only a proportion of trypanosome cytochrome is present in the holo-state (note the intensity of the TMB stain for equine cytochrome *c* relative to the amount of protein loaded). This we ascribe to the absence of additional, exogenous heme from the LB medium used to culture our *E. coli* and/or low expression of soluble *Tb*KCCS relative to apo-*Tb*cytc from the pCDFDuet-1 expression plasmid. Potentially, an extremely faint detection of heme is detected in lane E6 for *E. coli* expressing Tb*CYTC* but not Tb*KCCS*; we suggest if TMB-staining is present it possibly reflects coordination of heme iron by the N-terminal hexa-histidine tag in the recombinant trypanosome cytochrome *c*.

**Cell culture and transfection.** *L. mexicana* (M379-T7Cas9) promastigotes were grown in HOMEM medium (Gibco) supplemented with 10 % heat inactivated fetal calf serum (Gibco) with 1 % Penicillin/Streptomycin (Sigma-Aldrich) at 25^o^C. Transfections were carried out using the 4D Nucleofactor^TM^ (Lonza).

Gene deletion studies of *Lm*KCCS were carried out using a CRISPR-Cas9 toolkit in *L. mexicana* promastigotes genetically modified for constitutive expression of Cas9 nuclease and T7 RNA polymerase (**15**). This was done by providing resistance cassettes using primers with homology sites of 30 nucleotides upstream (F: ACGTCGATTCGCACGACGTCCACAAGGAGAgtataatgcagacctgctgc) and downstream (R: CTTGGCCAGCGCTGCAGAAAGGGAAAGCGGccaatttgagagacctgtgc) of the break site and were amplified from pPLOT plasmids. Guide DNA primers were used as a template for the sgRNA induced break site (5’ gaaattaatacgactcactataggGGCGGTAATTGTGGCGGCAG

gttttagagctagaaatagc and 3’ gaaattaatacgactcactataggAGCGGTACCACACGAGCGCG

gttttagagctagaaatagc).

pNUS-GFPcN (**16**) was used for episomal expression of *Lm*KCCS or *Tb*HCCS, C-terminally tagged with GFP. For cloning *Lm*KCCS, the fragment was amplified using forward (cacttgtcaagcgaattccatatgATGGCGGGGGCGGCGTGG) and reverse primer (gctcatggtaccagatctcatatgCGAGTGAGGCGGTGCCGCTTC). For cloning *Tb*HCCS, the fragment was amplified using forward (cacttgtcaagcgaattccatatgATGTGGGTGAGGACATTCCTG) and reverse primer (gctcatggtaccagatctcatatgCGGTGCCGCATGGCATTT). The PCR products were cloned into pNUS-GFPcN using Gibson assembly (NEB) as per manufacturer’s instructions. Following confirmation of episomal *Lm*KCCS::GFP (EC-L in **Fig. 2D**) or *Tb*HCCS::GFP (EC-T in **Fig. 2E**) expression, CRISPR-Cas9 was again used for gene deletion of Lm*KCCS*. PCR was used as a diagnostic tool to confirm facilitated knock out (FKO) using the following primer sets P1, (forward primer) TGGGCAGCGAGTTCAAGAAT and (reverse primer) AAGCGGGCAGAACTTCATCA (amplicon size 0.62 kb); P2, TGTCATTGTGACAGTGC and AAGCGGGCAGAACTTCATCA (1.027 kb); P3, TGTCATTGTGACAGTGC and GCAGCAGGTCTGCATTATAC (0.178 kb); P4, TGGGCAGCGAGTTCAAGAAT and GCATCACCTTCACCCTCTCC (1.025 kb); P5, CGGGTGCAAATCACCCAATG and CCTCCGAGTGTCATCCATCG (0.294 kb); P6, CGGGTGCAAATCACCCAATG and GCATCACCTTCACCCTCTCC (1.088 kb). As the protein loading control for immunoblot analysis of *Lm*KCCS::GFP or *Tb*KCCS::GFP expression, polyclonal antibodies detecting oligopeptidase B (anti-OPB) (**17**) were used.

**Flow cytometry.** Logarithmic cells (~5x10^6^-10^7^ cells ml^-1^) were washed in PBS and resuspend in 70 % methanol for 30 minutes. Cell were pelleted and washed with PBS containing 10 µgml^-1^ propidium iodide and 10 µgml^-1^ RNAase A. Fluorescence was measured using the PE-Cy5-Lin channel on Cyan and analyses carried using Summit V4 software (Beckman Coulter). Gating included all singlet cells.

**KCCS localisation.** Live imaging of promastigotes expressing *Lm*KCCS::mNeonGreen was carried out using CyGEL^TM^ (Biostatus) to immobilize cells. Samples were imaged immediately using a Zeiss AxioObserver microscope with 488 and 405 nm lasers. Images were processed using Zen Black (Zeiss) and Microvolution^TM^ deconvolution software.

**REFERENCES**

1. Acestor N, Panigrahi AK, Ogata Y, Anupama A, Stuart KD. 2009. Protein composition of *Trypanosoma brucei* mitochondrial membranes. Proteomic*s* 9:5497-5508.
2. Niemann M, Wiese S, Mani J, Chanfon A, Jackson C, Meisinger C, Warscheid B, Schneider A. 2013. Mitochondrial outer membrane proteome of *Trypanosoma brucei* reveals novel factors required to maintain mitochondrial morphology. Mol Cell Proteomics 12:515-528.
3. Babbitt SE, Sutherland MC, san Francisco B, Mendez DL, Kranz RG. 2015. Mitochondrial cytochrome *c* biogenesis: no longer an enigma. Trends Biochem Sci 40:446-455.
4. Verissimo AF, Daldal F. 2014. Cytochrome *c* biogenesis system I: an intricate process catalysed by a maturase supercomplex? Biochim Biophys Acta 1837:989-998.
5. Simon J, Hederstedt L. 2011. Composition and function of cytochrome *c* biogenesis system II. FEBS J 278:4179-4188.
6. Saint-Marcoux D, Wollman FA, de Vitry C. 2009. Biogenesis of cytochrome *b*_6_ in photosynthetic membranes. J Cell Biol 185:1195-1207.
7. Mészáros B, Erdős G, Dosztányi Z. 2018. IUPred2A: context-dependent prediction of protein disorder as a function of redox state and protein binding. Nucleic Acid Res 46:W329-W337.
8. Mizianty MJ, Uversky V, Kurgan L. 2014. Prediction of intrinsic disorder in proteins using MFD2p. Methods Mol Biol 1137:147-162.
9. Katoh K, Standley DM. 2013. MAFFT multiple alignment software version 7: improvements in performance and sustainability. Mol Biol Evol 30:772-780.
10. Thomas PE, Ryan D, Levin W. 1976. An improved staining procedure for the detection of the peroxidase activity of cytochrome P-450 on sodium dodecyl sulfate polyacrylamide gels. Anal Biochem 75:168-176.
11. Barr I, Guo F. 2015. Pyridine hemochromagen assay for determining the concentration of heme in purified solutions. Bio Protoc 5:e1594.
12. Margoliash E, Lustgarten J. 1962. Interconversion of horse heart cytochrome *c* monomer and polymers. J Biol Chem 237:3397-3405.
13. Paul K-G. 1948. The stability of cytochrome *c* at extreme pH values. Acta Chem Scand 2:430-439.
14. Hirota S, Hattori Y, Nagao S, Taketa M, Komori H, Kamikubo H, Wang Z, Takahashi I, Negi S, Sugiura Y, Kataoka M, Higuchi Y. 2010. Cytochrome *c* polymerization by successive domain swapping at the C-terminal helix. Proc Natl Acad Sci USA 107:12854-12859.
15. Beneke T, Madden R, Makin L, Valli J, Sunter J, Gluenz E. 2017. A CRISPR Cas9 high-throughput genome editing toolkit for kinetoplastids. R Soc Open Sci 4:170095.
16. Tetaud E, Lecuix I, Sheldrake T, Baltz T, Fairlamb AH. 2002. A new expression vector for *Crithidia fasciculata* and *Leishmania*. Mol Biochem Parasitol 120:195-204.
17. Munday JC, McLuskey K, Brown E, Coombs GH, Mottram JC. 2011. Oligopeptidase B deficient mutants of *Leishmania major*. Mol Biochem Parasitol 175:49-57.
