# Supplementary material for: Divergent Cytochrome c Maturation System in Kinetoplastid Protists
Source: mBio. 2021 May 4;12(3):e00166-21. doi: 10.1128/mBio.00166-21 (PMC8262978; doi:10.1128/mBio.00166-21)
Supplement: FIG S1 [file mbio.00166-21-sf001.pdf]

Extreme divergence of the kinetoplastid cytochrome *c* maturation system

Asma Belbelazi, Rachel Neish, Martin Carr, Jeremy C. Mottram, and Michael L. Ginger

```
Tb                                                 MWVRTFLRLCGCKSPNAAAI  20
Ad                        MLGRTIFRQCSKHATKATVGSLLQSEANKNLNNNHNEESAVAA  43
Lm
Bs                                                 MLRRSLRRLCNSLDAATAGG  20
Tbrr           MFLQSLLRRCAANNKGPTNIGKGVMGSCNTTGISKPVAGSSSPSSVASVSNTNTAAPCTD  60
Pk_sp                     MRRSVLRFGSVGAAWADSIFRTKKGDVESSMSEPQPDKLPTAS  43


Tb         TSGSSWMTAAAWASLGSEFSSVSESKFLQQVPDGFLTSRATT-DMMPAEQLLLSMVEENE  79
Ad         HSGTGWLAGAAWASLGSEFQTVEVDKFLKPLPDHFLTPRATT-DIEPQEELLHSLVEQNE  102
Lm               MAGAAWASLGSEFKNVAEDKFLKPVPDQFLTPRATT-DIQPAEELLSKLVEENA  53
Bs         AAGSGWMTGAAYASIGSEFRNVDAEKFLLPKPDSFLSPRATT-DVMPAEVLMSQFVEENE  79
Tbrr       GSAPGWMDGASWASLGAEFQGVKEDKFLLPKPNGFLSVRATT-DVQPAEEVLQSLVQHNE  119
Pk_sp      VKQSPFVEVETTESLD-EVEVYQPDDEVKPEPTP--KPTFNTEMFLSKEKILAHEADIIR  100
                 :   :  *:. *.     .. :   *    .   .*  .   * ::   .:

Tb         ERYKGVDVRDPSSMAVY----EGERPRWMTLGGQVRAVSEFVSGHLCHHISLPAWKELFD  135
Ad         KMYEGIDVRDPSSLAVY----EGERPRWLTMGGQVRAVSEFISGHLCHHISLPEWKLLFD  158
Lm         ERYKGIDVRDPSSMAIY----EGERPRWMTMGGQVRAVSEFISGHLCHHISLPEWKDLFD  109
Bs         ERYKGIDVRDPNSLAHY----DGEKPSWLTLGAQVTAVSEFISGHLTHHVALEEWKQLFD  135
Tbrr       DMYKGLDVTDPNSMVHF----DGERPTWMTLGDQVRAVSEFISGHLVHHIALEAWKELFD  175
Pk_sp      KTYDGVDVRSPSTLPANHLFPDAVPQRKLTWRDQVLAVSDFMSGHLAHHVMLDEWAKLLD  160
           . *.*:** .*.::       :.     :*   ** ***:*:**** **: *  *  *:*

Tb         LQYAEMDLTYWLYVLHVHMVSRRATSVPIEKFNRRREVLEEILLTMFDSWAATSEDVMGR  195
Ad         LQYAEMDLTYWLYVLHVHLVSRRATAIPIESFARRREVLEELLLTLFDSWASTSEDIMGR  218
Lm         LEYAEMDLTYWLYVLHVHLVSRRATSIPIEKFNRRREVLEELLVTMFDGWAATSEDIMGR  169
Bs         LKYAEMDLTYWLYVIHVHILARRATSVPIEKFNRRREVLEEVLLTMFDSWAYTSEDIMGR  195
Tbrr       LKYIEMDLVYWLYVIHLHIISRRATSVKIENWHRRREVMEEMLFTMFDSWAHTSEEIMGR  235
Pk_sp      LETLDVEIRYFIWILHLQMISRRSLAIPVENWARRREVLQEMQASMRSSWEESCTQVLGR  220
           *:  :::: *:::::*:::::**: :: :*.: *****::*:  :: ..*  :. :::**

Tb         PPLNKIRFYIKDMYYVTAVNFEEAL--------LHDG--------------PGADLMLLG  233
Ad         PPLNKIKYYIRDMYYVTAVNFEEAL--------LHDG--------------AGADLMLMG  256
Lm         PPLNKIKYYIRDMYYVTAVNFEEAL--------LHDG--------------PGADMMLMG  207
Bs         PPLQKIRFYIKDMYYVTAVNFEEAL--------LHDG--------------AGADLMLLG  233
Tbrr       PPLNKIRHYIKDMYYVTAVNFEEAL--------LHDG--------------PGADLMLFG  273
Pk_sp      PPPQRQKDYLRDMYLVVAMNFEEALSGTEINASLSDSKEIVPSKGDGKDAASGSDLALMS  280
           ** :: : *::*** *.*:******                         . *:*: *:.

Tb         FLMKFCPLPRPEDVPLYTYYSLVHYIRFHTALLDRIPDESIAKGNFNFLSPTDPRIFEQY  293
Ad         FLMKFCPLPRPEDVPMYTYYTLVHYIRFHTALFDRMSDESFAKGNFNFLSPTDPIIFEKY  316
Lm         FLMKFCPLPRPEDVPMFTYYTLVHYVRFHTALFDRIPDEEFAKGNFNFLSPTDPLIFSKY  267
Bs         FLMKFCPLSRPEDIPIYTYYTLVHYIRFHVALFDRIPDESIAKGNFNFLSPTHSAIFEPY  293
Tbrr       FLVKFCPLPRPEDIPVYTYFNLVHYIRFHTALFDRIPDEMISKGNFSFLSPNDPAITKKY  333
Pk_sp      FLFRFLPFQRPEDIPMYSYYRLVHYIRFHLALLDRISDEDVSKGNFNFVNPLSEAICENY  340
           **.:* *: ****:*:::*: ****:*** **:**: ** .:****.*:.*    * . *

Tb         SEVTLDQVIRSWTVEASEEEVKCHAAP                           320
Ad         SEIAYDDVIKSWTVEEEGNHTNSNNNDNHNNSHDSETEKKE             357
Lm         SDIAYDEVIRGWTVQEGEDGGGEAAPPHS                         296
Bs         SDVALDDVIRGWTADNYWEEMKAQKESGGDNNTNDTTTGHQHGGEEGGEKRP  345
Tbrr       TEIEFDEVIRSWKVSEDNDATE                                355
Pk_sp      QDIPLR                                                346
            ::
```

FIG S1 KCCS is a conserved kinetoplastid protein. Ad, *Angomonas deanei* (EPY32355.1); Bs, *Bodo saltans* (CUF09763.1); Lm, *Leishmania mexicana* (XP_003872427.1); Pk_sp, *Perkinsela* (KNH04224.1); Tb, *Trypanosoma brucei* (XP_843981.1); Tbrr, *Trypanoplasma borreli* (). Genbank accession numbers for the sequences used in the alignment are provided in the parentheses.
