# Supplementary material for: Divergent Cytochrome c Maturation System in Kinetoplastid Protists
Source: mBio. 2021 May 4;12(3):e00166-21. doi: 10.1128/mBio.00166-21 (PMC8262978; doi:10.1128/mBio.00166-21)
Supplement: FIG S2 [file mbio.00166-21-sf002.pdf]

Extreme divergence of the kinetoplastid cytochrome *c* maturation system

Asma Belbelazi, Rachel Neish, Martin Carr, Jeremy C. Mottram, and Michael L. Ginger

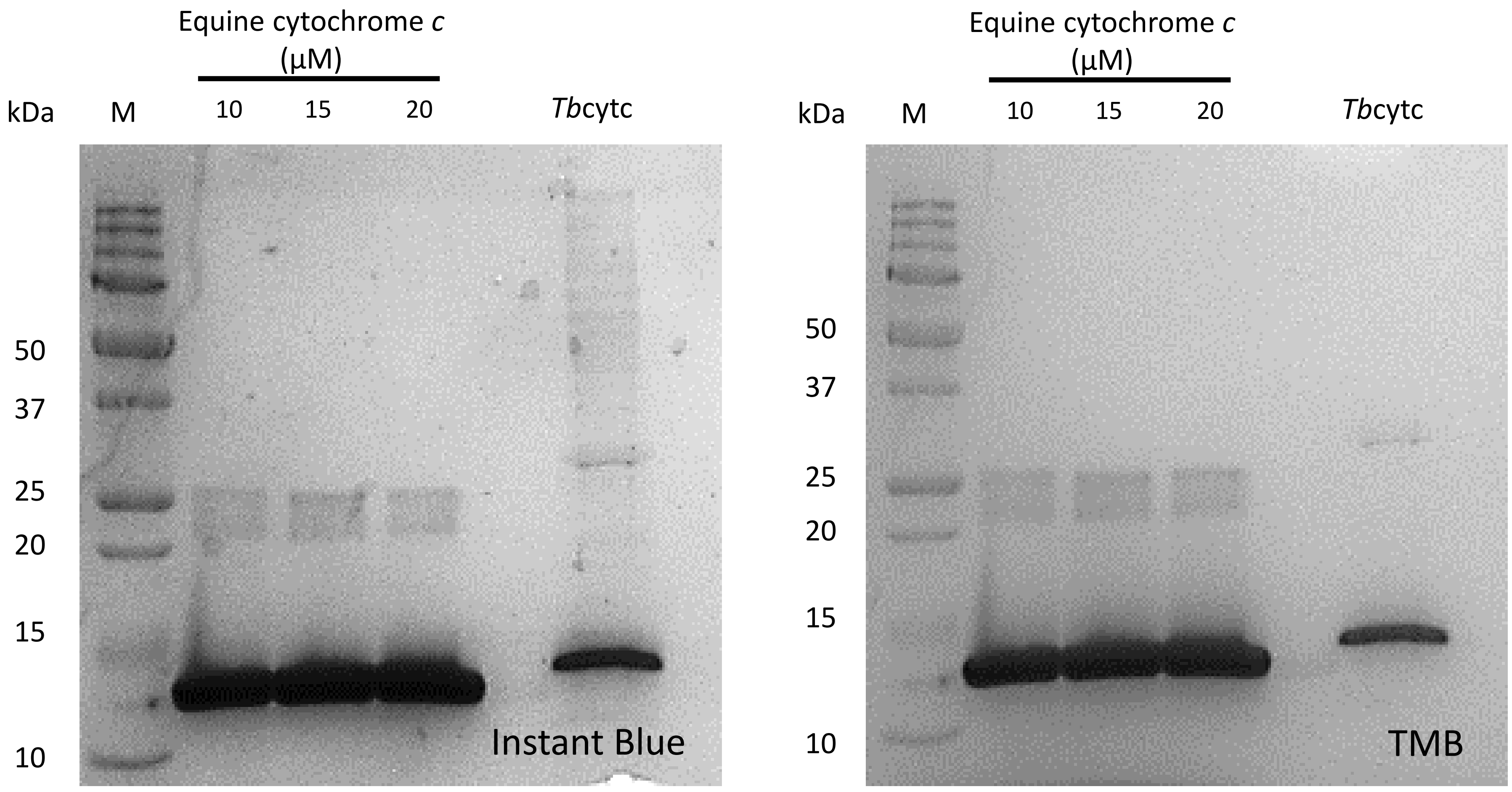

FIG S2 Purity of and covalent heme attachment to *Tb*^{His}CYTC purified for uv/vis spectroscopy. *Tb*^{His}CYTC was purified from 8 l of *E. coli* induced for recombinant expression of *Tb*^{His}CYTC and *Tb*KCCS as described in Text S1 and concentrated to 0.5 ml using a Vivaspin-20 centrifugal concentrator with a m.w. cut-off of 3 kDa. 1/250th of the purified protein was taken without acetone precipitation for analysis by SDS-PAGE under non-reducing conditions. Duplicate 12% polyacrylamide gels were stained with either Instant Blue (to confirm purity) or 3,3′,5,5′-tetramethylbenzidine (to detect covalent attachment of heme to protein). Equine holocytochrome *c* was loaded as indicated.
